# Supplementary material for: Highly Extensible Physically Crosslinked Hydrogels for High‐Speed 3D Bioprinting
Source: Adv Healthc Mater. 2025 Feb 16;14(10):2404988. doi: 10.1002/adhm.202404988 (PMC12004426; doi:10.1002/adhm.202404988)
Supplement: Supplementary file 1 — Supporting Information [file ADHM-14-0-s001.pdf]

ADVANCED HEALTHCARE MATERIALS

Supporting Information

for *Adv. Healthcare Mater.*, DOI 10.1002/adhm.202404988

Highly Extensible Physically Crosslinked Hydrogels for High-Speed 3D Bioprinting

*Ye Eun Song, Noah Eckman, Samya Sen, Carolyn K. Jons, Olivia M. Saouaf and Eric A. Appel**

Supporting Information

Highly extensible physically crosslinked hydrogels for high-speed 3D bioprinting

Ye Eun Song, Noah Eckman, Samya Sen, Carolyn K. Jons, Olivia M. Saouaf, Eric A. Appel

Supplementary Figures

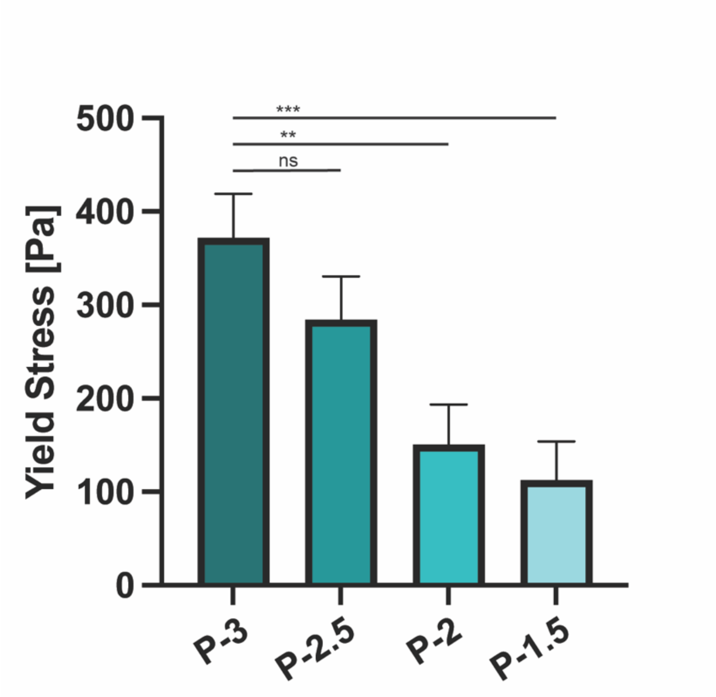

Figure S1. Yield stress of HPMC-C_{18} hydrogel formulations. One-way ANOVA was used for multiple comparisons. Significance levels are indicated as $**p < 0.01$, and $***p < 0.001$.

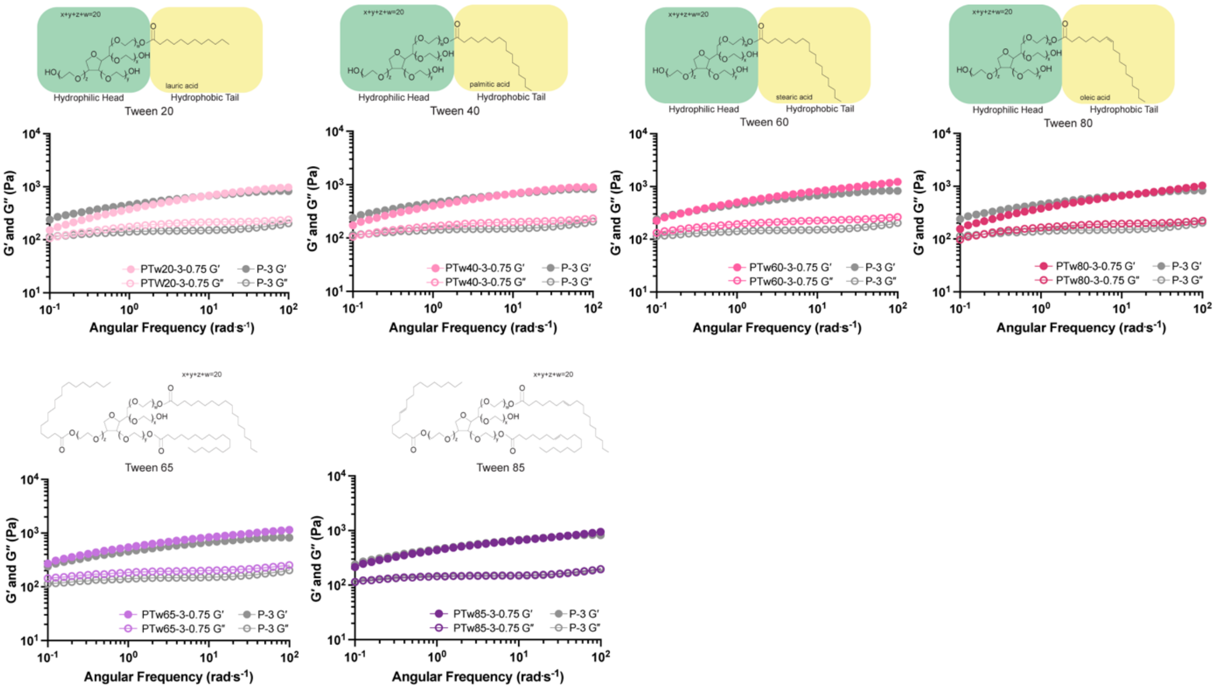

Figure S2. Frequency-dependent shear rheology of various HPMC-C_{18} hydrogel formulations comprising different tween surfactants.

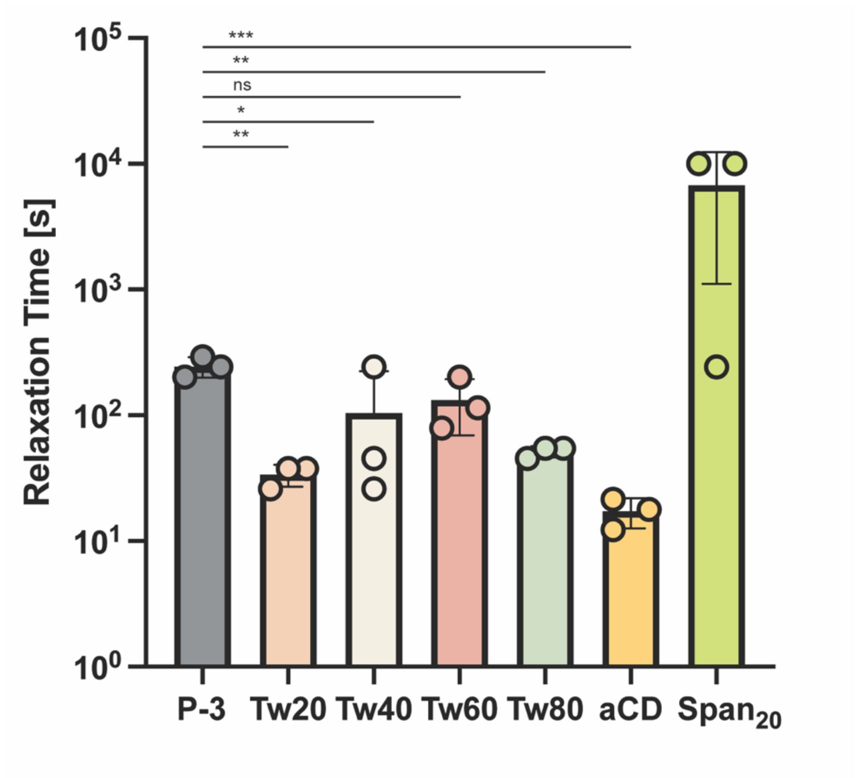

Figure S3. Relaxation time for various HPMC-C_{18} hydrogel formulations comprising different tween surfactants. One-way ANOVA was used for multiple comparisons. Significance levels are indicated as $*p < 0.05$, $**p < 0.01$, and $***p < 0.001$.

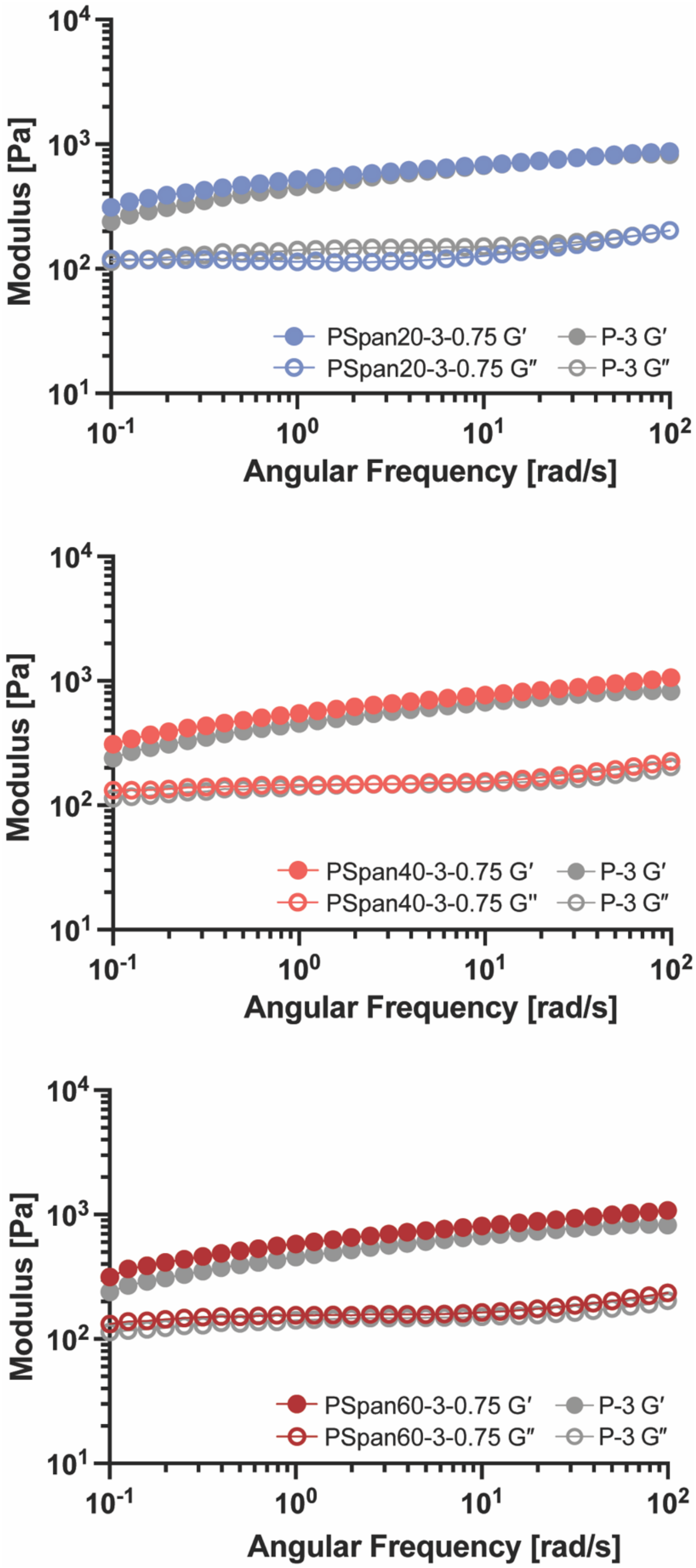

Figure S4. Frequency-dependent shear rheology of various HPMC-C_{18} hydrogel formulations comprising different Span surfactants.

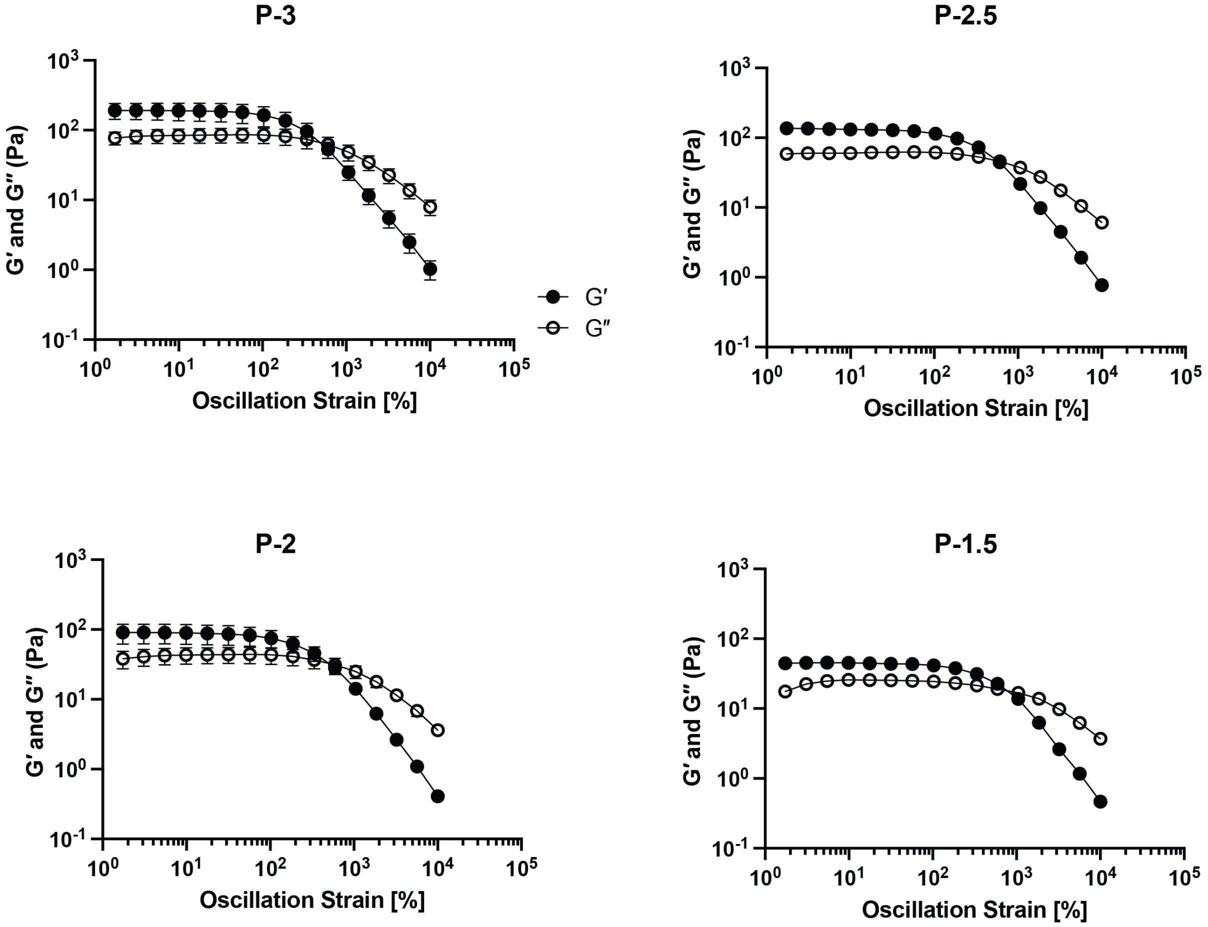

Figure S5. Amplitude-dependent shear rheology of various HPMC-C_{18} hydrogel formulations.

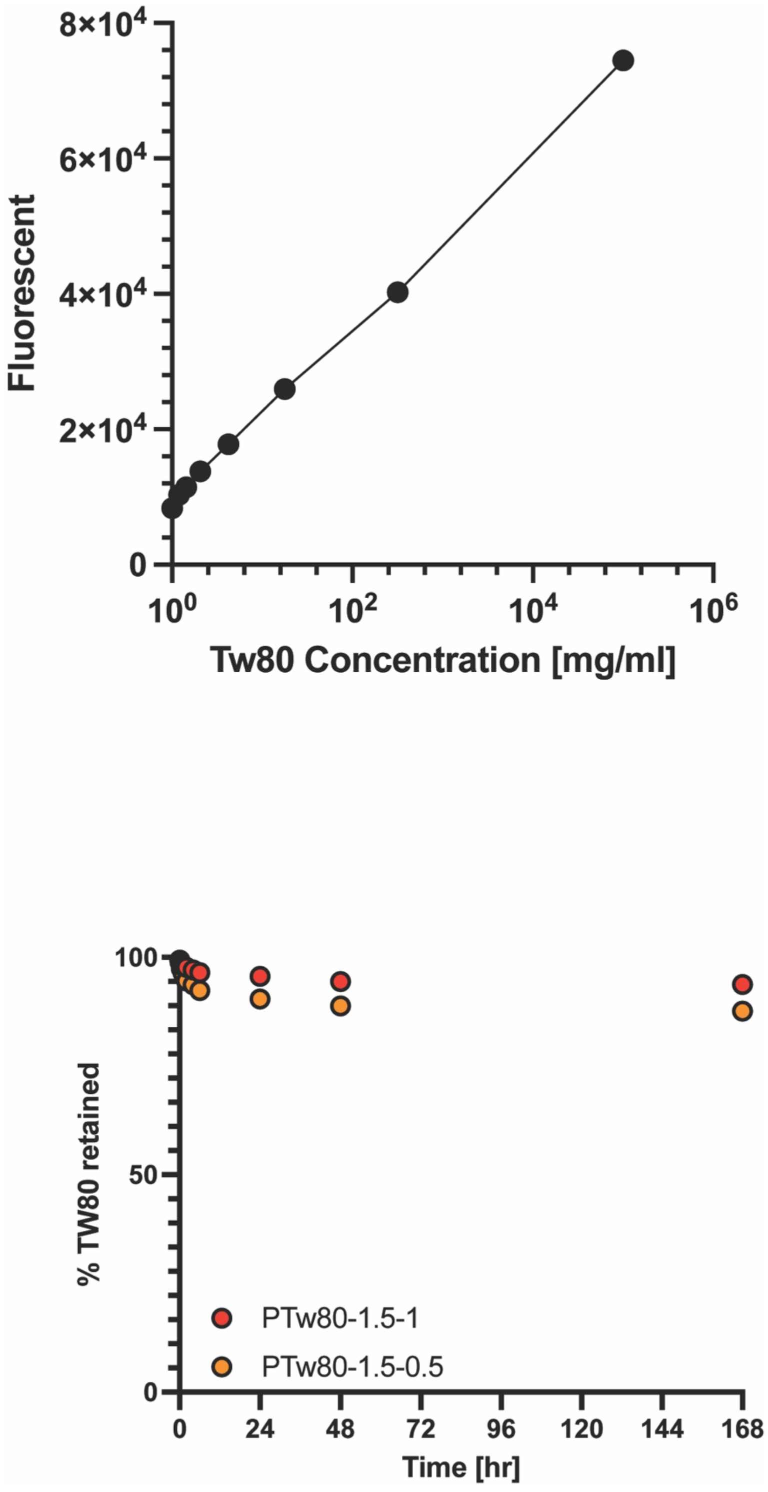

Figure S6. Retention of Tw80 in PTw80 hydrogels observed with *in vitro* release assays.

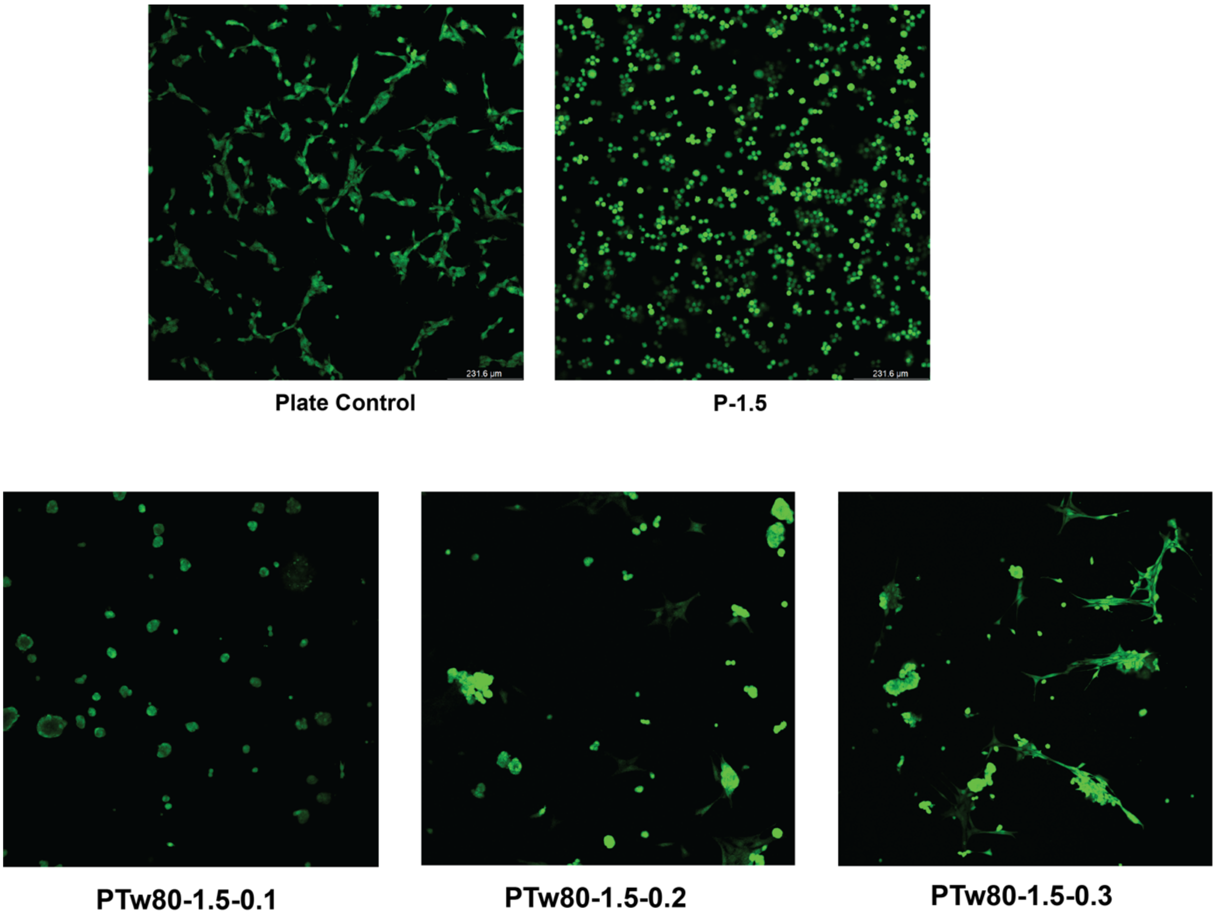

Figure S7. Confocal images of cells entrapped in PTw80 hydrogels.

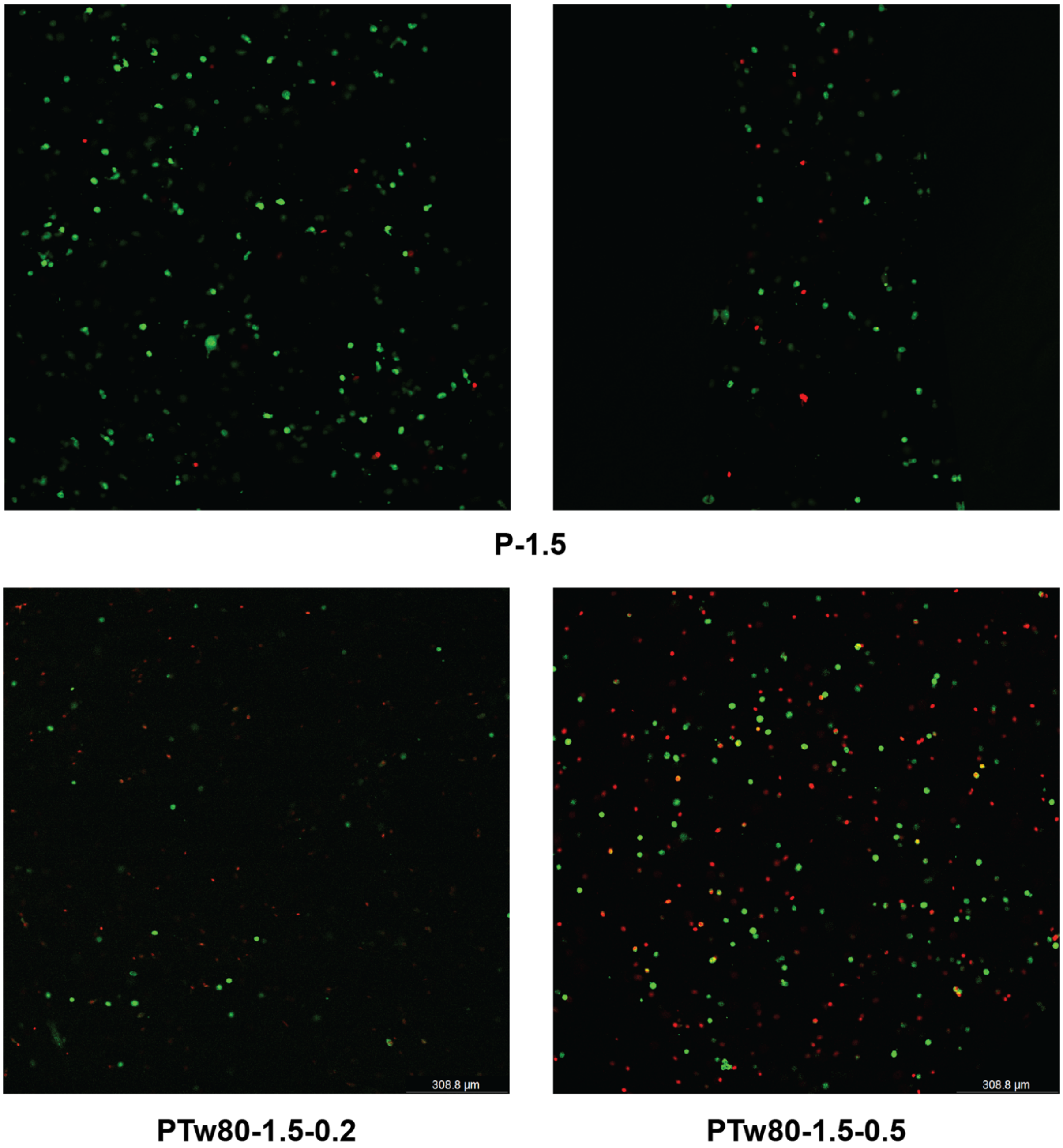

Figure S8. Confocal images of cells entrapped in PTw80 hydrogels comprising different amounts of Tw80 post printing.

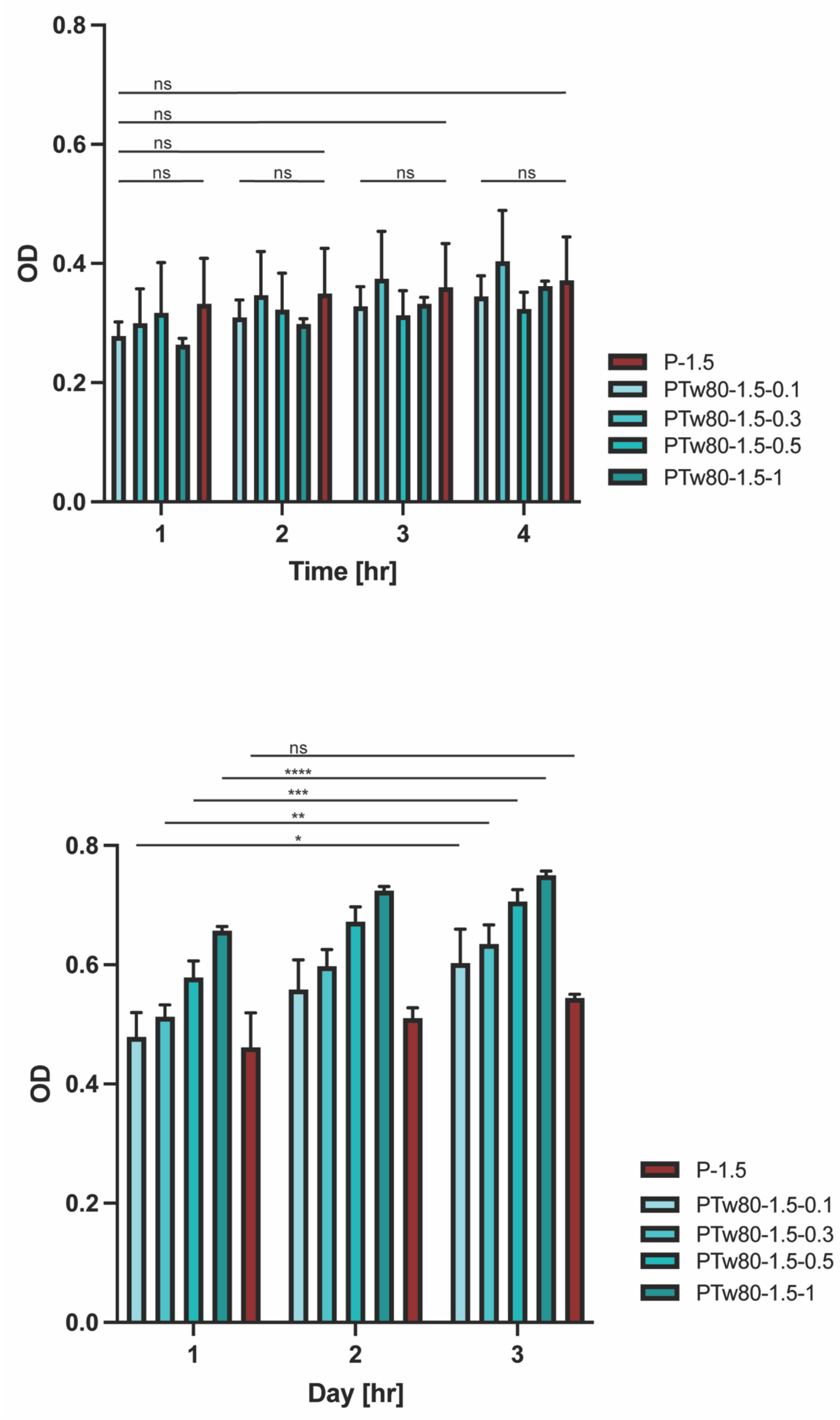

Figure S9. Cell counting kit-8 assay of PTw80 hydrogels. Two-way ANOVA was used for multiple comparisons. Significance levels are indicated as $*p < 0.05$, $**p < 0.01$, $***p < 0.001$, and $****p < 0.0001$.

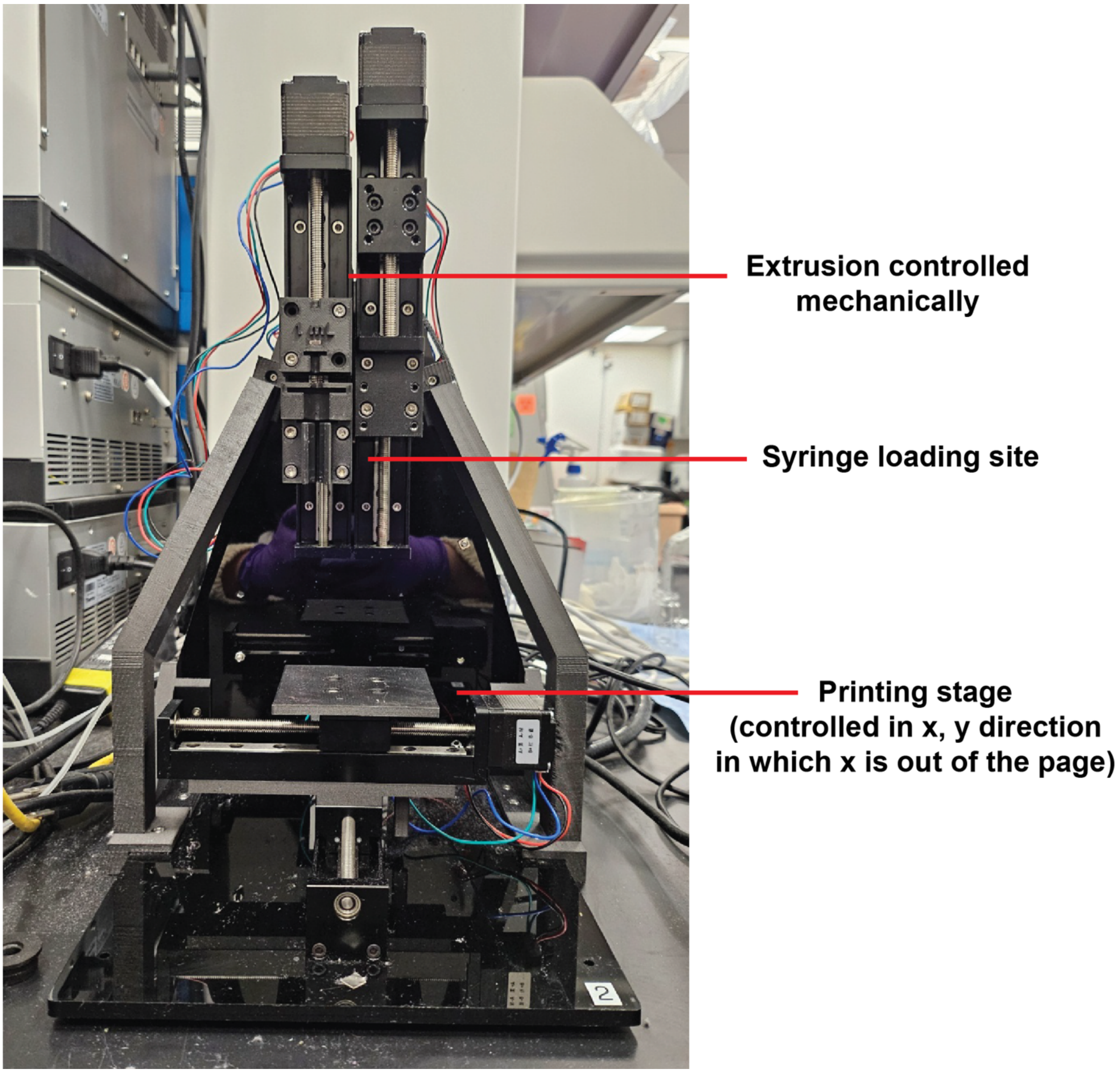

Figure S10. Extrusion-based 3D printer image.

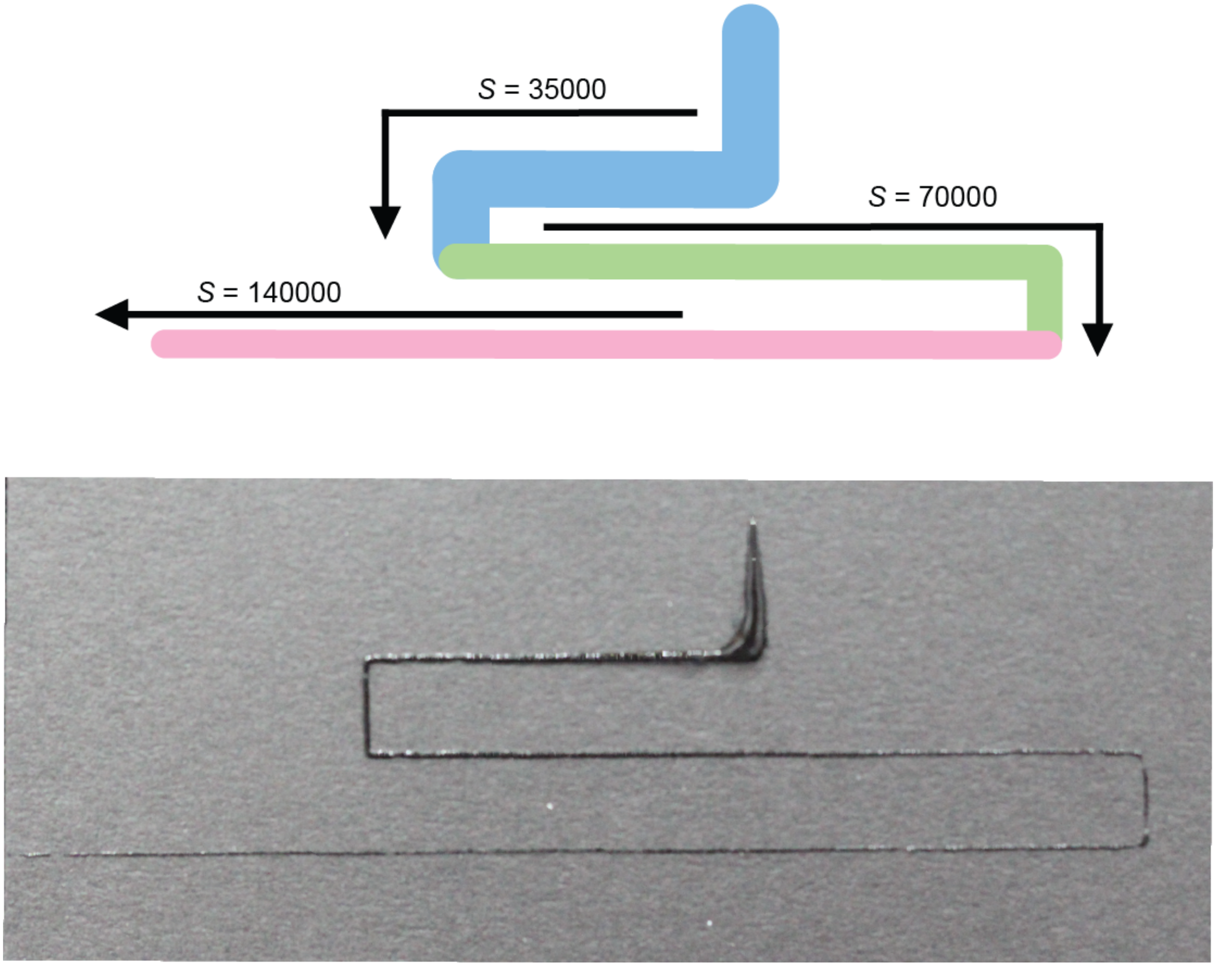

Figure S11. Schematic of 3d printing experiment for PTw80-1.5-1 and representative printed image.

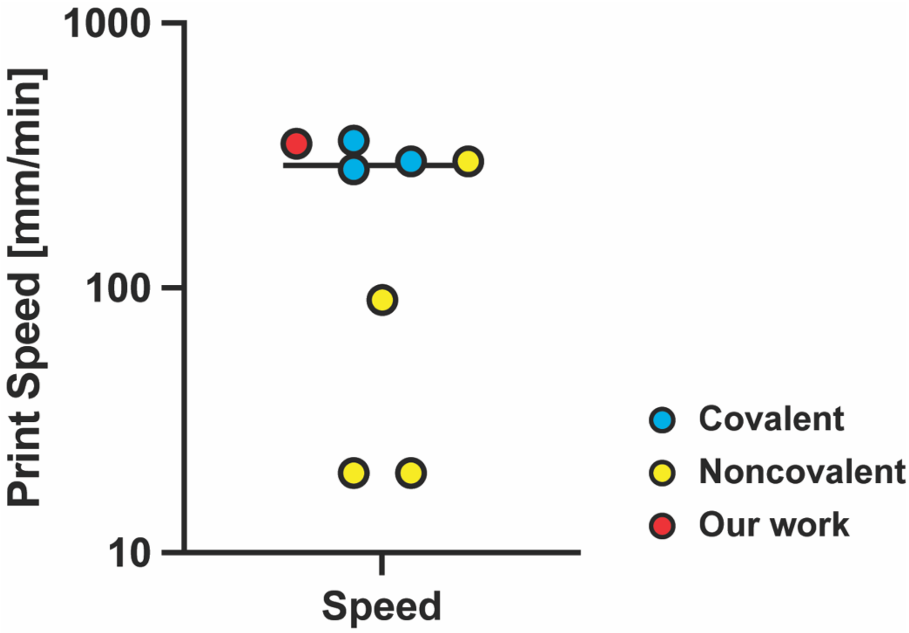

Figure S12. Absolute print speed (mm/min) comparison with hydrogel-bioinks in extrusion-based printing system reported in current literatures

Supplementary Videos

Video S1. Example filament stretching extensional rheometry (FiSER) of PTw80-1.5-1 at 1800s^{-1}.
